# *Xylella* Genomics and Bacterial Pathogenicity to Plants

**DOI:** 10.1002/1097-0061(200012)17:4<263::AID-YEA44>3.0.CO;2-G

**Published:** 2000

**Authors:** J. M. Dow, M. J. Daniels

**Affiliations:** 1 The Sainsbury Laboratory John Innes Centre Norwich Research Park Colney Norwich NR4 7UH UK

## Abstract

*Xylella fastidiosa*, a pathogen of citrus, is the first plant pathogenic bacterium for which the complete genome sequence has been published. Inspection of the sequence reveals high relatedness to many genes of other pathogens, notably *Xanthomonas campestris*. Based on this, we suggest that *Xylella* possesses certain easily testable properties that contribute to pathogenicity. We also present some general considerations for deriving information on pathogenicity from bacterial genomics.


**Yeast**
*Yeast* 2000; **17**: 263–271.

**Review Article**

### *Xylella* genomics and bacterial pathogenicity to plants

J. M. Dow and M. J. Daniels*
*The Sainsbury Laboratory, John Innes Centre, Norwich Research Park, Colney, Norwich NR4 7UH, UK*

*Correspondence to:
M. J. Daniels, The Sainsbury Laboratory, John Innes Centre, Norwich Research Park, Colney, Norwich NR4 7UH, UK.
E-mail: mike.daniels@bbsrc.ac.uk

Received: 21 September 2000
Accepted: 21 September 2000

### Abstract

***Xylella fastidiosa*, a pathogen of citrus, is the first plant pathogenic bacterium for which the complete genome sequence has been published. Inspection of the sequence reveals high relatedness to many genes of other pathogens, notably *Xanthomonas campestris*. Based on this, we suggest that *Xylella* possesses certain easily testable properties that contribute to pathogenicity. We also present some general considerations for deriving information on pathogenicity from bacterial genomics. Copyright © 2000 John Wiley & Sons, Ltd.**

**Keywords: *Xylella fastidosa*; *Xanthomonas campestris*; gene disruption; phenotype assessment; secretion; regulation; signalling; enzymes; polysaccharide**

### Introduction

Since the publication of the complete genome sequence of *Haemophilus influenzae* in 1987, rapid progress has been made in bacterial genomics and today approximately 100 bacterial genomes have been or are about to be completed. Many of the organisms chosen are human or animal pathogens. It is not surprising that funding agencies have supported work on human pathogens because functional genomics provides new strategies for the discovery of urgently needed antibacterial therapeutic agents. Despite the obvious importance of human pathogens, it should not be forgotten that plant pathogens can also be indirect agents of human misery. It is generally believed that, globally, up to 20% of potential crop yields are lost because of disease. Bacterial plant diseases are most severe in the tropics, where their effects can be locally catastrophic. Apart from exploitation of genetically resistant crops (if they exist), few satisfactory control measures are available. The need to devise new control strategies based on understanding pathogenicity is a compelling argument for supporting sequencing projects. The publication of the first complete genome sequence of a bacterial phytopathogen, *Xylella fastidiosa* (*Xf*), by a consortium of researchers in Brazil [39] is therefore to be welcomed. The choice of organism is interesting. It appears to have been driven by economic considerations: *Xf* is a major pathogen of citrus trees in Brazil. As its name suggests, *Xf* is a difficult microorganism to handle. It has hitherto been largely ignored by molecular geneticists, who have preferred to work with more tractable genera, such as *Erwinia, Pseudomonas, Ralstonia* and *Xanthomonas*. The genomes of members of these genera are typically twice the size of the *Xf* genome.

Analysis of the *Xf* genomic sequence has revealed a number of genes whose products have very high amino acid sequence relatedness to gene products in *Xanthomonas* spp., in particular *Xanthomonas campestris* pv. *campestris* (hereafter called *X. c. campestris*). Many of these *Xanthomonas* genes have established roles either in virulence or in the biosynthesis of the extracellular polysaccharide xanthan, which as well as being an important industrial product is believed to be a virulence determinant. To some extent amino acid sequence relatedness between *Xf* and *Xanthomonas* is not particularly surprising, since these bacterial species are closely related [46]. However, the high degree of relatedness strongly suggests that the *Xanthomonas* and *Xf* genes are orthologous. In contrast, the relative size of the genomes of *Xf* ($\sim$2.7 Mb) and *Xanthomonas* ($\sim$5.5 Mb) indicates that there are distinct differences in the complement of genes carried by the two bacteria. *Xf* may represent a

Copyright © 2000 John Wiley & Sons, Ltd.

'minimal' plant pathogen. In addition to its plant hosts, *Xf* can colonize tissues of its sharpshooter insect vector. However, it is not pathogenic to the insects and thus differs from *Pseudomonas aeruginosa*, certain strains of which are polyphyletic pathogens [12,32] able to cause disease in mammals, plants and invertebrates. Although this paper only discusses plant pathogenesis, studies of *Xf* may also reveal determinants of bacteria–insect interaction specificity.

The differences in genome size between *Xf* and *X. c. campestris* probably reflect the different disease strategies and growth capabilities of the two organisms. *Xf* depends on leafhoppers for its transmission and in plants is limited to the xylem. *X. c. campestris* can infect intact plants and is capable of growth and survival on plant leaf surfaces as well as in soil. In the following sections we highlight some of the similarities (and differences) between gene products and gene organization in *Xanthomonas* and *Xf* that may inform studies of *Xf* pathogenesis. We also consider general strategies for the functional genomics of plant pathogens.

### *Xylella* has no type III secretion system

A principal difference between *Xf* and *Xanthomonas* spp. is the absence in *Xf* of a type III secretion system, which in other plant pathogenic bacteria is encoded by genes within *hrp* gene clusters. Type III secretion systems are widely distributed in phytopathogenic bacteria, where they function to deliver effectors of disease into plant cells (for recent reviews, see [16,23]). Some of these virulence determinants may act to suppress plant defence responses and hence promote disease, whereas others may act to promote the release of nutrients from the plant cell. In some cases, however, secreted effectors can be recognized by the plant to trigger plant defence. In this case they are considered to be avirulence determinants. Examination of the *Xf* genome revealed no gene product with amino acid sequence relatedness to the products of known avirulence genes in other plant pathogenic bacteria. The lack of dependence of *Xf* pathogenesis on a type III secretion system may reflect the insect-mediated transmission, vascular restriction and slow growth of the bacterium. For example, pathogenesis by *Xf* may not require the suppression of specific processes within living plant cells. It should be noted, however, that the bacterium may be able to kill plant cells through the action of plant cell wall degrading enzymes (see below) and haemolysin-like toxins. In contrast to the situation in *Xf*, type III secretion systems are required for the pathogenesis of *X. c. campestris* and *X. oryzae* pv. *oryzae* [1,49], vascular pathogens that colonize the xylem elements in crucifers and rice, respectively. These bacteria enter intact plants through hydathodes, structures located at the leaf margin at the ends of the veins. Having colonized the hydathodes, the bacteria gain access to the xylem elements, where they proliferate. At later stages of disease, bacteria can be released from the vascular system to invade the surrounding mesophyll tissue. The type III-secreted effectors could clearly have a role in each of these phases of the disease cycle.

### Type II protein secretion in *Xylella* and *Xanthomonas*

*X. c. campestris* synthesizes a number of extracellular enzymes that are capable of degrading components of the plant extracellular matrix (cell wall), including pectin, cellulose, proteins and glycoproteins [8]. The export of these extracellular enzymes (and perhaps other proteins) from the bacterial cell is achieved via a type II secretion system encoded by the cluster of 11 genes, *xpsEFGHIJKLMND* [11,25]. Type II secretion is signal sequence-dependent, the pathway is the main terminal branch of the general secretion pathway [31,34]. The type II secretion pathway is required for virulence in both *X. c. campestris* and *X. o. oryzae* [9,33]. The same gene organization as found in *X. c. campestris* occurs in *Xf* and most of the gene products (XF1517–1527) have very high amino acid sequence relatedness to their *Xanthomonas* counterparts. The *Xf* type II secretion system is probably involved in the export of cell wall degrading enzymes, including endoglucanase (XF0810, XF0818, XF2708), polygalacturonase (XF2466) and perhaps several proteases (XF2330, XF0267, XF1851, XF0816). The occurrence of several isoforms of endoglucanase and protease is consistent with observations of many plant pathogenic microorganisms. One possible function of these enzymes in *Xf* pathogenesis is to degrade the pit membranes, allowing bacteria to move into previously uncolonized xylem vessels. Other roles

Copyright © 2000 John Wiley & Sons, Ltd. 

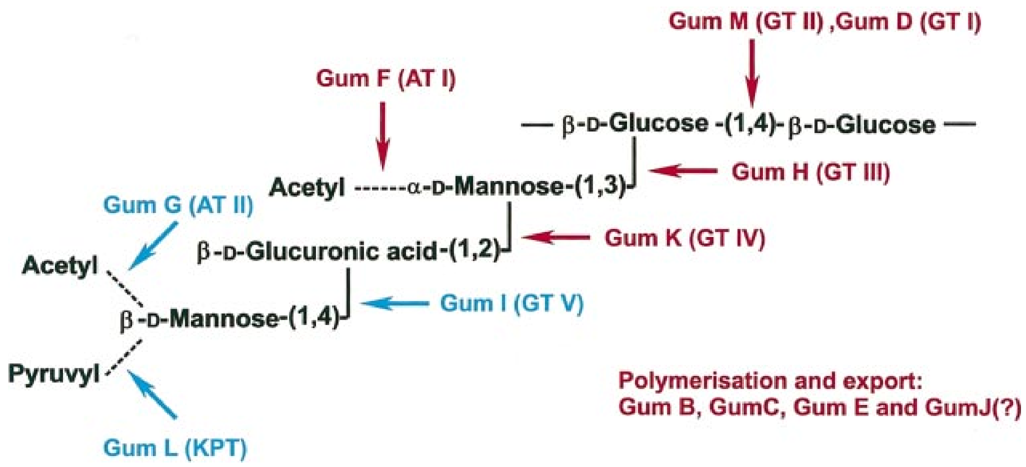

**Figure 1.** The structure of the repeating pentasaccharide unit of xanthan *of Xanthomonas campestris* together with the proposed functions of the *gum* gene products involved its assembly, decoration with acetyl and pyruvyl residues, polymerization and export from the bacterial cell. Dashed lines indicate that the repeating unit is variably decorated; the terminal mannose residue carries either an acetyl or pyruvyl moiety. Abbreviations: GT, glycosyl transferase; AT, acetyl transferase; KPT, ketal pyruvate transferase. Those *gum* gene products that are common to *Xylella* and *Xanthomonas* are indicated in red; those that are only found in *Xanthomonas* are shown in blue may be to mobilize cell walls for nutritional purposes or to overcome plant defences. The putative polygalacturonase precursor from *Xf* is highly related to the enzyme from *Ralstonia solanaceraum*, the causal agent of bacterial wilt. The polygalacturonase from *R. solanacearum* is required for the full virulence of this vascular pathogen to tomato [37].

### Extracellular polysaccharides

Many plant pathogenic bacteria produce extracellular polysaccharides that contribute to the ability of these organisms to cause disease on plants [7]. In *X. c. campestris*, the extracellular polysaccharide is xanthan, which is also an important commercial product. Xanthan is a polymer of repeating pentasaccharide units with the structure mannose-($\beta$-1,4)-glucuronic acid-($\beta$-1,2)-mannose-($\alpha$-1,3)-cellobiose [42]. The pentasaccharide units are derivatized with acetyl and pyruvyl moieties. Biosynthesis of xanthan is believed to occur in at least two stages. In the first, the repeating unit is sequentially assembled linked to a polyprenol through a diphosphate bridge. In the second stage, the repeating units are polymerized and the polymer liberated to the growth medium. The genes that encode the enzymes involved in the transfer of the sugars and the non-glycosidic constituents are located in a cluster that comprises 12 predicted open reading frames, *gumB* to *gumM*. The function of some of the gene products has been established, whereas others have only been suggested [18,21,26,44]. GumD, GumH, GumI, GumK and GumM are known to be involved in the assembly of the pentasaccharide lipid intermediate. Gum L is the pyruvate ketal transferase. GumB, GumC, GumE and GumJ are speculated to have a role in the polymerization and translocation of xanthan. The *Xf* genome contains genes with highly related products with the gene order *gumBCDEFHJKM* (XF2370–XF2360). Two short ORFs (XF2363 and XF2368) are also present in this region. However, *gumI* (encoding the transferase which incorporates the terminal mannose), *gumL* and *gumG* (encoding one of two acetyl transferases) were missing. BLAST searches of the *Xf* genome revealed no other gene products with amino acid sequence relatedness to GumL or GumI. These results suggest that *Xf* may make a modified polysaccharide that lacks the terminal mannose residues and is not pyruvylated (Figure 1), although this has not been experimentally determined. Mutants of *X. c. campestris* carrying non-polar insertions in *gumI* are also able to produce a polytetrasaccharide, although only to 10% of the amount of polymer produced by the wild-type [21]. Significantly, *gumI*

Copyright © 2000 John Wiley & Sons, Ltd. 

mutants of *X. c. campestris* are not altered in their virulence when inoculated into the petioles of cabbage plants [21]. Mutants of *X. c. campestris* with non-polar insertions into *gumK* can synthesize a lipid-linked trisaccharide *in vitro* but produce only very low amounts of a polytrisaccharide polymer *in vivo* [21]. These modified polysaccharides may have particular properties that make them useful in biotechnological or other industrial applications [42]. One limitation of the study and exploitation of some of these modified polymers is the low level of production, which may be in part a consequence of the substrate specificity of the polymerization/export apparatus for the different lipid-linked oligosaccharides. The apparent natural variation in the polysaccharides produced by *Xf* and *X. c. campestris* may reflect differences in the substrate specificities for lipid–oligosaccharide precursors that could be exploited to elevate the production of modified xanthans in *X. c. campestris*.

### The regulation of the synthesis of virulence determinants

The production of extracellular enzymes and extracellular polysaccharide by *X. c. campestris* is strictly regulated both during growth in liquid media and during disease. Regulation probably occurs as an adaptation to particular environmental changes. Work in our own laboratory has identified a cluster of genes that act to regulate the synthesis of these virulence factors. This cluster, which we have called *rpf* (for regulation of pathogenicity factors), comprises nine genes (*rpfA-I*) and is located within a 21.9 kb region of the *X. c. campestris* chromosome [2,10,43,48]. The left-hand part of this region of the chromosome comprises six contiguous *rpf* genes with the gene order *rpfABFCHG*. Transposon insertion in any of these genes leads to coordinate downregulation of synthesis of all extracellular enzymes and EPS. The remaining *rpf* genes (*rpfD, rpfE, rpfI*), which are located to the right of *rpfA-G*, are grouped with genes of diverse function. Transposon insertions in these latter *rpf* genes lead to only minor effects (*rpfD*) or to complex effects on only certain enzymes (*rpfE, rpfI*).

In some cases, sequence analysis of the *rpf* genes and/or biochemical studies of the *rpf* mutants have given a guide to possible function of the gene products. *rpfA* encodes the major aconitase of *X. c. campestris* and is implicated in iron homeostasis [48]. *rpfB* encodes a long chain fatty acyl CoA ligase and, together with *rpfF*, is involved in regulation mediated by a small diffusible molecule, which we have called DSF (for diffusible signal factor) [2]. Neither *rpfF* nor *rpfB* mutants are able to make DSF, although *rpfF* mutants can be phenotypically corrected for the production of extracellular enzymes and EPS by the exogenous addition of DSF or by growth on plates in proximity to wild-type strains. RpfF has limited amino acid sequence relatedness to enoyl CoA hydratase and we have speculated that RpfF and RpfB are involved in diverting intermediates of lipid metabolism to DSF production [2].

*rpfC* encodes a hybrid two-component regulator, containing both sensor kinase and response regulator domains, and contains an additional C-terminal phosphorelay (HPt) domain [40,43]. *rpfC* is in an operon with *rpfH* and *rpfG* [40]. The predicted protein RpfG has a regulatory input domain attached to a specialized version of an HD domain, suggested previously to function in signal transduction [17]. The predicted protein RpfH is structurally related to the sensory input domain of RpfC. RpfG, RpfH and RpfC are proposed to participate in a signal transduction system linking perception of environmental signals, including perhaps DSF itself, to the activation of pathogenicity gene expression and to the negative regulation of DSF synthesis [40]. The predicted proteins, RpfD, RpfE and RpfI, show the highest amino acid sequence relatedness to hypothetical proteins from *Caulobacter crescentus*, *Bordetella pertussis* and *Klebsiella pneumoniae*, respectively [10].

Remarkable synteny exists between the region of the chromosome of *X. c. campestris* that contains the *rpf* gene cluster and that region of the *Xf* genome encoding products XF1108–XF1115 (Figure 2). Furthermore, the amino acid sequence similarities between all of the genes which are represented in both chromosomes is very high; in BLASTP comparisons E values were all less than $10^{-99}$ and homologies exist throughout the length of the proteins. Genes encoding RpfA (XF0290) and RpfB (XF0287), which are immediately to left of *rpfF* in *X. c. campestris*, are located in a different part of the *Xf* genome although they are closely linked. In contrast, BLASTP searches revealed no gene products in *Xf* with significant amino acid

Copyright © 2000 John Wiley & Sons, Ltd. 

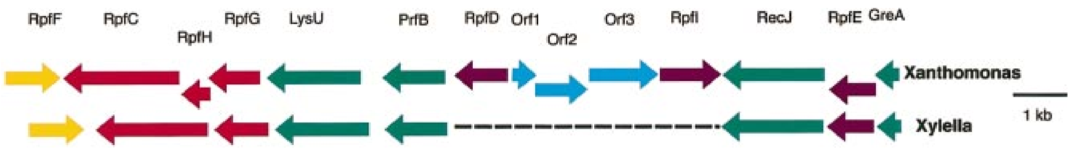

**Figure 2.** Synteny between the *rpf* gene clusters of *Xylella fastidiosa* and *Xanthomonas campestris*. The *rpf* genes of *X. c. campestris*, which regulate the synthesis of pathogenicity factors, are clustered in a region of the chromosome that also contains genes with broader regulatory functions and others of diverse function. Rec J is a single-stranded DNA-specific exonuclease, GreA is a transcriptional elongation factor, PrfB is peptide release factor 2, LysU is lysyl tRNA synthetase. The dashed line indicates that *recJ* and *prfB* are contiguous in the *Xf* genome sequence similarity to RpfD, Orf1, Orf2, Orf3 or RpfI.

### Intercellular signalling

The extremely high degree of sequence relatedness between gene products implicated in the synthesis and perception of DSF in *X. c. campestris* and predicted gene products from *Xf* provides strong circumstantial evidence for the existence of a DSF regulatory system in *Xf*. It is increasingly evident that cell–cell signalling mediated by diffusible molecules plays a large role in regulating diverse physiological processes, including virulence to plants and animals, across distant genera of bacteria [20,22,35]. Notably, cell–cell signals enable bacteria to behave in a group fashion, coordinating changes in gene expression in response to the changing environment and growing cell population, a phenomenon which has been called 'quorum sensing'. The most common signalling molecules found among Gram-negative bacteria, including plant pathogens, are *N*-acyl derivatives of homoserine lactone (*N*-AHLs) but other molecules such as lipids, peptides and $\gamma$-butyrolactones are also found [14,15,35]. *X. c. campestris* has apparently evolved a novel system for cell–cell signalling. DSF is not an *N*-AHL and a recent survey has indicated that production of compounds with *N*-AHL activity is very rare in *Xanthomonas* species [4]. A second low molecular weight signalling molecule in *X. c. campestris* that has overlapping functions with DSF has been tentatively characterized as a butyrolactone [5,28,29,30].

Proteins of the LuxI and LuxR families are involved in the synthesis of *N*-AHLs and in quorum sensing-mediated gene regulation, respectively, in many bacteria [15]. Homology searches (BLASTP) with LuxI family members from a number of bacterial species, including *R. solanacearum* and *Erwinia* spp., did not reveal any significant hits in *Xf*. There were also no gene products with significant sequence relatedness to a number of proteins unrelated to LuxI implicated in the synthesis of autoinducers or diffusible signal molecules in several other bacteria. These included LuxM and LuxL, which are implicated in the synthesis of *N*-AHLs in *Vibrio harveyi* [3], LuxS, which is responsible for autoinducer synthesis in *Escherichia coli* and *Salmonella typhimurium* [41], and PhcB, which is responsible for the synthesis of hydroxypalmitic acid methyl ester, a regulator of synthesis of extracellular enzymes and extracellular polysaccharide in *R. solanacearum* [13,36].

### What is the physiological state of xylem-infecting bacteria?

Electron micrographs of infected plants often show high local concentrations of bacteria in the tissues. Micrographs of *Xf*-infected citrus can be seen on the Brazilian group's web site (**http://onsona.lbi.ic.unicamp.br/xf/**). There are many similar examples in the literature, including a study of cabbage infected with *X. c. campestris* [45]. The implications of such pictures are often overlooked. We calculate that the concentration of *X. c. campestris* in xylem vessels is at least $10^{13}$ cells/ml; for comparison, a dense stationary phase laboratory broth culture contains about $10^9$ cells/ml. It is instructive to consider the bacteria as growing in a continuous-flow system such as a chemostat, fed by the flow of xylem sap. The flow rate of nutrients through xylem vessels can be estimated [19,38] and, making the most optimistic assumptions about the ability of bacteria to sequester nutrients from sap, it is clear that the rate of increase of bacterial biomass must be very small, much less than one doubling in 24 h. The conclu-

Copyright © 2000 John Wiley & Sons, Ltd. 

sion is that bacteria in infected plants are subject to severe nutritional stress and hence mechanisms employed by bacteria to respond to and protect against physiological stress will be important in pathogenicity. The processes by which bacteria accumulate to such high levels merit further research.

### Strategies for functional genomics of plant pathogens

Although *Xf* is the only published phytopathogen genome sequence, several others are in progress. These include *Xanthomonas axonopodis* pv. *citri* (Brazil, by the consortium responsible for *Xf*), *X. c. campestris* (China), *X. o. oryzae* (Japan), *R. solanacearum* (France), *P. syringae* pv. *tomato* (USA), *E. carotovora* (Finland) and *Agrobacterium tumefaciens* (USA). This set includes examples of the major taxonomic, physiological and economically important pathogens. It is timely to consider how functional studies could be implemented to yield information on pathogenicity mechanisms. The Brazilian consortium has already established a programme for functional analysis of *Xf* (http://www.lbm.fcav.unesp/br/fun/). The portfolio of projects highlights some of the disadvantages of using an 'unpopular' organism such as *Xf*. Many of the necessary techniques, such as gene disruption, that have been used routinely for other pathogens for many years, will have to be developed from scratch. Methodology used for studying bacterial pathogenicity is described in two books [24,47]; here we briefly consider approaches to evaluating the role of sequenced genes in bacteria–plant interactions.

### Gene expression

The concept that some 'pathogenicity' genes might show differential expression in *X. c. campestris* inhabiting host tissues was proposed in 1987 [27] and the approach used was the forerunner of '*in vivo* expression technology' (IVET). Comparisons of bacterial gene expression levels have mainly used gene fusion technology. However, the availability of complete sequences of organisms invites the use of microarrays for genome-wide surveys. It is not yet clear what technical obstacles will have to be surmounted in order to measure bacterial transcript levels directly in infected plants. Experimental manipulation of expression levels can yield useful information on gene function. Apart from relatively crude methods, such as multicopy cloning, no general methods are available for influencing bacterial gene expression in infected plants.

### Gene disruption

Procedures for gene disruption based on either direct transposon mutagenesis or recombinational exchange between the genome and suitably altered cloned sequences have been developed for all the major genera of plant pathogens. The use of marked and unmarked and in-frame deletions is particularly informative.

### Phenotype assessment

The 'pre-genomic era' of molecular phytobacteriology relied heavily on isolation of mutants that produced altered disease symptoms. The need to screen large numbers of individuals dictated the use of simple, rapid and unsophisticated techniques for testing pathogenicity. The disadvantage of simple methods is that the results of pathogenicity tests may depend on how the inoculation of plants is carried out [6]. As an example, *rpfC* mutants of *X. c. campestris* produce symptoms indistinguishable from the wild-type if infiltrated directly into leaf panels, but inoculation into vein endings (which more closely resembles the natural infection route) shows them to have much reduced virulence [40]. The feasibility constraints imposed by mass screening programmes no longer apply when evaluating the phenotype of specific mutants and more refined and time-consuming methods become acceptable. Based on experience with many classes of *X. c. campestris* mutant, we suggest the following set of phenotypic tests of mutants as a starting point for evaluating the role of a gene in plant–bacterial interactions:

(i) Cultural characteristics (auxotrophy, quantitative growth properties, sensitivity to physicochemical factors, substrate utilization, motility and chemotaxis).
(ii) Production and/or secretion of factors considered to be candidate pathogenicity factors (extracellular enzymes, polysaccharides, toxins).
(iii) Cell surface and other structural changes (altered surface polymers, attachment to surfaces, biofilm formation).

Copyright © 2000 John Wiley & Sons, Ltd. 

(iv) Changes to the transcriptome, proteome and metabolome (i.e. global effects of regulatory genes).
(v) Ability to survive or grow on external plant surfaces, in the rhizosphere, etc.
(vi) Ability to enter and colonize stomata, hydathodes, etc.
(vii) Ability to spread within plants (parenchyma, vascular tissue, etc.).
(viii) Ability to multiply in plant tissues.
(ix) Disease symptoms produced after inoculation by appropriate routes.
(x) Ability to incite a hypersensitive response in resistant or non-host plants.
(xi) Changes in host range.
(xii) Ability to induce various plant defence responses.

### From genomics to disease control?

In many discussions on the use of genomic information for the development of therapeutic agents against human pathogens, a five-step strategy has been proposed:

(i) Identification of key virulence factors by functional genomics research.
(ii) Development of biochemical assays for the function of the virulence factors.
(iii) Screening chemical libraries for lead compounds which inhibit the function.
(iv) Testing activity of lead compounds in experimental model infections.
(v) Development of therapeutic agents from lead compounds.

In principle, an analogous strategy could be adopted for plant pathogens. Chemical control of fungal diseases has been highly effective but, for a variety of reasons, bacterial diseases of plants have, historically, not been satisfactorily controlled by chemicals (an exception is those pathogens, such as *Xf*, which need insect vectors and for which insecticides may disrupt the disease cycle). For other bacteria, the most effective control measure, apart from use of good agronomic practices, is the deployment of genetic resistance to pathogens. At present the search for new sources of genetic resistance to incorporate into crop breeding programmes requires large-scale glasshouse and field inoculation with bacteria and measurement of disease development. It is to be expected that functional genomics will lead to the identification of previously unsuspected bacterial virulence factors and mechanisms. This should in turn permit the development of biochemical probes and assays to reveal natural genetic variation in susceptibility of targets in plants or to guide the manipulation of targets in the laboratory.

### Acknowledgements

The Sainsbury Laboratory is supported by the Gatsby Charitable Foundation. Work in the authors' laboratory has also been supported by the BBSRC (Grants 83/PRS12153 and P05167).

### References

1. Arlat M, Gough CL, Barber CE, Boucher C, Daniels MJ. 1991. *Xanthomonas campestris* contains a cluster of *hrp* genes related to the larger *hrp* cluster *of Pseudomonas solanacearum*. *Mol Plant Microbe Interact* **4**: 593–601.
2. Barber CE, Tang J-L, Feng J-X, Pan MQ, Wilson TJG, Slater H, *et al.* 1997. A novel regulatory system required for pathogenicity of *Xanthomonas campestris* is mediated by a small diffusible signal molecule. *Mol Microbiol* **24**: 555–566.
3. Bassler BL, Wright ME, Showalter RE, Silverman MR. 1993. Intercellular signalling in *Vibrio harveyi*: sequence and function of genes regulating expression of luminescence. *Mol Microbiol* **9**: 773–786.
4. Cha C, Gao P, Chen YC, Shaw PD, Farrand SK. 1998. Production of acyl-homoserine lactone quorum sensing signals by Gram-negative plant-associated bacteria. *Mol Plant Microbe Interact* **11**: 1119–1129.
5. Chun W, Cui J, Poplawsky A. 1997. Purification, characterization and biological role of a pheromone produced by *Xanthomonas campestris* pv. *campestris*. *Physiol Mol Plant Pathol* **51**: 1–14.
6. Daniels MJ. 1998. Testing pathogenicity. In *Bacterial Pathogenesis* (Methods in Microbiology, vol 27), Williams P, Ketley J, Salmond G (eds). Academic Press: London; 129–137.
7. Denny TP. 1995. Involvement of bacterial polysaccharides in plant pathogenesis. *Ann Rev Phytopathol* **33**: 173–197.
8. Dow JM, Daniels MJ. 1994. Pathogenicity determinants and global regulation of pathogenicity of *Xanthomonas campestris* pv. *campestris*. In *Bacterial Pathogenesis of Plants and Animal*, Dangl JL (ed.). Springer-Verlag: Berlin; 29–41.
9. Dow JM, Scofield G, Trafford K, Turner PC, Daniels MJ. 1987. A gene cluster in *Xanthomonas campestris* pv. *campestris* required for pathogenicity controls the excretion of polygalacturonate lyase and other enzymes. *Physiol Mol Plant Pathol* **31**: 261–271.
10. Dow JM, Feng J-X, Barber CE, Tang J-L, Daniels MJ. 2000. Novel genes involved in the regulation of pathogenicity factor production within the *rpf* gene cluster of *Xanthomonas campestris*. *Microbiology* **146**: 885–891.

Copyright © 2000 John Wiley & Sons, Ltd. 

11. Dums F, Dow JM, Daniels MJ. 1991. Structural characterization of protein secretion genes of the bacterial phytopathogen *Xanthomonas campestris* pathovar *campestris*-relatedness to secretion systems of other Gram-negative bacteria. *Mol Gen Genet* **229**: 357–364.
12. Finlay RB. 1999. Bacterial disease in diverse hosts. *Cell* **96**: 315–318.
13. Flavier AB, Clough SJ, Schell MA, Denny TP. 1997. Identification of 3-hydroxypalmitic acid methyl ester as a novel autoregulator controlling virulence in *Ralstonia solanacearum*. *Mol Microbiol* **26**: 251–259.
14. Fuqua C, Winans SC, Greenberg EP. 1994. Quorum sensing in bacteria: the LuxR–LuxI family of cell density-responsive transcriptional regulators. *J Bacteriol* **176**: 269–275.
15. Fuqua C, Winans SC, Greenberg EP. 1996. Census and consensus in bacterial ecosystems: the LuxR–LuxI family of quorum-sensing transcriptional regulators. *Ann Rev Microbiol* **50**: 727–751.
16. Galan J, Collmer A. 1999. Type III secretion machines: bacterial devices for protein delivery into host cells. *Science* **284**: 1322–1328.
17. Galperin MY, Natale DA, Aravind L, Koonin EV. 1999. A specialized version of the HD hydrolase domain implicated in signal transduction. *J Mol Microbiol Biotechnol* **1**: 303–305.
18. Ielpi L, Couso RO, Dankert MA. 1993. Sequential assembly of the polyprenol-linked pentasaccharide repeating unit of the xanthan polysaccharide in *Xanthomonas campestris*. *J Bacteriol* **175**: 2490–2500.
19. Jeschke WD, Pate JS. 1991. Modelling of the uptake, flow and utilization of C, N and $H_2O$ within whole plants of *Ricinus communis* L based on empirical data. *J Plant Physiol* **137**: 488–498.
20. Kaiser D, Losick R. 1993. How and why bacteria talk to each other. *Cell* **73**: 873–885.
21. Katzen F, Ferreiro DU, Oddo CG, *et al*. 1998. *Xanthomonas campestris* pv. campestris *gum* mutants: effects on xanthan biosynthesis and plant virulence. *J Bacteriol* **180**: 1607–1617.
22. Kell DB, Kaprelyants AS, Grafen A. 1995. Pheromones, social-behavior and the functions of secondary metabolism in bacteria. *Trends Ecol Evol* **10**: 126–129.
23. Kjemtrup S, Nimchuk Z, Dangl JL. 2000. Effector proteins of phytopathogenic bacteria: bifunctional signals in virulence and host recognition. *Curr Opin Microbiol* **3**: 73–78.
24. Klement Z, Rudolph K, Sands DC. 1990. *Methods in Phytobacteriology*. Akademiai Kiado: Budapest.
25. Lee HM, Wang KC, Liu YL, *et al*. 2000. Association of the cytoplasmic membrane protein XpsN with the outer membrane protein XpsD in the type II secretion apparatus of *Xanthomonas campestris* pv. *campestris*. *J Bacteriol* **182**: 1549–1557.
26. Marzocca MP, Harding NE, Petroni EA, Cleary JM, Ielpi L. 1991. Location and cloning of the ketal pyruvate transferase gene of *Xanthomonas campestris*. *J Bacteriol* **173**: 7519–7524.
27. Osbourn AE, Barber CE, Daniels MJ. 1987. Identification of plant-induced genes of the bacterial pathogen *Xanthomonas campestris* pathovar *campestris* using a promoter-probe plasmid. *EMBO J* **6**: 23–28.
28. Poplawsky AR, Chun W. 1997. *pigB* determines a diffusible factor needed for extracellular polysaccharide slime and xanthomonadin production in *Xanthomonas campestris* pv. *campestris*. *J Bacteriol* **179**: 439–444.
29. Poplawsky AR, Chun W. 1998. *Xanthomonas campestris* pv. *campestris* requires a functional *pigB* for epiphytic survival and host infection. *Mol Plant Microbe Interact* **11**: 466–475.
30. Poplawsky AR, Chun W, Slater H, Daniels MJ, Dow JM. 1998. Synthesis of extracellular polysaccharide extracellular enzymes and xanthomonadin in *Xanthomonas campestris*: evidence for the involvement of two intercellular regulatory signals. *Mol Plant Microbe Interact* **11**: 68–70.
31. Pugsley AP, Francetic O, Possot OM, Sauvonnet N, Hardie KR. 1997. Recent progress and future directions in studies of the main terminal branch of the general secretory pathway in Gram-negative bacteria – a review. *Gene* **192**: 13–19.
32. Rahme LG, Ausubel FM, Cao H, *et al*. 2000. Plants and animals share functionally common bacterial virulence factors. *Proc Natl Acad Sci U S A* **97**: 8815–8821.
33. Ray SK, Rajeshwari R, Sonti RV. 2000. Mutants of *Xanthomonas oryzae* pv. *oryzae* deficient in general secretory pathway are virulence deficient and unable to secrete xylanase. *Mol Plant Microb Interact* **13**: 394–401.
34. Russel M. 1998. Macromolecular assembly and secretion across the bacterial cell envelope: type II protein secretion systems. *J Mol Biol* **279**: 485–499.
35. Salmond GPC, Bycroft BW, Stewart G, Williams P. 1995. The bacterial enigma: cracking the code of cell–cell communication. *Mol Microbiol* **16**: 615–624.
36. Schell MA. 1996. To be or not to be: how *Pseudomonas solanacearum* decides whether or not to express virulence genes. *Eur J Plant Pathol* **102**: 459–469.
37. Schell MA, Roberts DP, Denny TP. 1988. Analysis of the *Pseudomonas solanacearum* polygalacturonase encoded by *pglA* and its involvement in phytopathogenicity. *J Bacteriol* **170**: 4501–4508.
38. Schurr U. 1998. Xylem sap sampling- new approaches to an old topic. *Trends Plant Sci* **3**: 293–298.
39. Simpson AJG, Reinach FC, Arruda P, *et al*. 2000. The genome sequence of the plant pathogen *Xylella fastidiosa*. *Nature* **406**: 151–157.
40. Slater H, Alvarez-Morales A, Barber CE, Daniels MJ, Dow JM. 2000. A two-component system involving an HD–GYP domain protein links cell–cell signalling to pathogenicity gene expression in *Xanthomonas campestris*. *Mol Microbiol* (in press).
41. Surette MG, Miller MB, Bassler BL. 1999. Quorum sensing in *Escherichia coli*, *Salmonella typhimurium*, and *Vibrio harveyi*: a new family of genes responsible for autoinducer production. *Proc Natl Acad Sci U S A* **96**: 1639–44.
42. Sutherland JW, Xanthan. In *Xanthomonas*, Swings JG, Civerolo EL (eds). Chapman and Hall: London; 363–388.
43. Tang JL, Liu YN, Barber CE, Dow JM, Wootton JC, Daniels MJ. 1991. Genetic and molecular analysis of a cluster of *rpf* genes involved in positive regulation of synthesis of extracellular enzymes and polysaccharide in *Xanthomonas campestris* pathovar *campestris*. *Mol Gen Genet* **226**: 409–417.
44. Vojnov AA, Zorreguieta A, Dow JM, Daniels MJ, Dankert MA. 1998. Evidence for a role for the *gumB* and *gumC* gene products in the formation of xanthan from its pentasaccharide repeating unit by *Xanthomonas campestris*. *Microbiology* **144**: 1487–1493.

Copyright © 2000 John Wiley & Sons, Ltd. 

45. Wallis FM, Rijkenberg FHJ, Joubert JJ, Martin MM. 1973. Ultrastructural histopathology of cabbage leaves infected with *Xanthomonas campestris*. *Physiol Plant Pathol* **3**: 371–378.
46. Wells JM, Raju BC, Hung HY, Weisburg WG, Mandelco-paul L, Brenner DJ. 1987. *Xylella fastidiosa* gen. nov, sp. nov- Gram-negative, xylem-limited, fastidious plant bacteria related to *Xanthomonas* spp. *Int J Syst Bacteriol* **37**: 136–143.
47. Williams P, Ketley J, Salmond G. 1998. *Bacterial Pathogenesis* (Methods in Microbiology, vol.27). Academic Press: London.
48. Wilson TJG, Bertrand N, Tang J-L, *et al.* 1998. The *rpfA* gene of *Xanthomonas campestris* pathovar *campestris*, which is involved in the regulation of pathogenicity factor production, encodes an aconitase. *Mol Microbiol* **28**: 961–970.
49. Zhu WG, Magbanua MM, White FF. 2000. Identification of two novel *hrp*-associated genes in the *hrp* gene cluster of *Xanthomonas oryzae*. *J Bacteriol* **182**: 1844–1853.

Copyright © 2000 John Wiley & Sons, Ltd. 

## References

[PDF_00590] Barber C. E., Tang J. L., Feng J. X., Pan M. Q., Wilson T. J., Slater H., Dow J. M., Williams P., Daniels M. J. (1997). A novel regulatory system required for pathogenicity of Xanthomonas campestris is mediated by a small diffusible signal molecule.. Mol Microbiol.

[PDF_00696] Pugsley A. P., Francetic O., Hardie K., Possot O. M., Sauvonnet N., Seydel A. (1997). Pullulanase: model protein substrate for the general secretory pathway of gram-negative bacteria.. Folia Microbiol (Praha).

[PDF_00737] Tang J. L., Liu Y. N., Barber C. E., Dow J. M., Wootton J. C., Daniels M. J. (1991). Genetic and molecular analysis of a cluster of rpf genes involved in positive regulation of synthesis of extracellular enzymes and polysaccharide in Xanthomonas campestris pathovar campestris.. Mol Gen Genet.

